# Acculturation Stress, Satisfaction, and Frustration of Basic Psychological Needs and Mental Health of Chinese Migrant Children: Perspective from Basic Psychological Needs Theory

**DOI:** 10.3390/ijerph18094751

**Published:** 2021-04-29

**Authors:** Qiang Ren, Shan Jiang

**Affiliations:** Department of Sociology, Zhejiang University, Hangzhou 310058, China; renq@zju.edu.cn

**Keywords:** acculturation stress, basic psychological needs, mental health, migrant children, China

## Abstract

Acculturation stress is prevalent among migrant populations. The current study examines whether acculturation stress influences migrant children’s mental health through the mediators of the satisfaction and frustration of basic psychological needs for autonomy, relatedness, and competence. A sample of 484 migrant children is obtained in Kunming, China using a multi-stage cluster random sampling. Data are analyzed through structural equation modeling in Mplus 8.0. Results indicate that acculturation stress has a direct impact on children’s depression but no significant direct effect on children’s happiness. Acculturation stress also has indirect effects on depression and happiness via the mediators of need satisfaction and frustration. Acculturation stress is negatively associated with need satisfaction and positively associated with need frustration, which is further significantly predictive of children’s happiness and depression. Overall, this study validates the basic psychological needs theory in the context of China’s internal migration. Findings contribute to the understanding of the mechanisms that underlie the relationship between acculturation stress and psychological outcomes and provide practical implications for future interventions.

## 1. Introduction

In the past four decades, the increasing number of migrant children in China became a widespread concern in the country’s economic transformation and urbanization. Data from the National Health Commission of China (2018) showed that the Chinese migrant population reached 244 million in 2017, comprising approximately 17% of the total population [1]. The All-China Women’s Federation (2013) reported that the total amount of Chinese migrant children was 35.81 million, which increased by 41.37% from 2005 to 2013 [2]. On the basis of the Hukou Policy (household registration system), migrant children without an urban Hukou are regarded as secondary citizens in the host city [3] and are incapable of obtaining the same rights, such as education and medical care, as their local counterparts [4].

Under such an unfair institutional arrangement, migrant children are vulnerable and may experience migration-related stress [5,6,7]. Therefore, adjusting to new environments and rebuilding their lives in host cities is arduous [8,9]. Acculturation stress causes a high incidence of mental health problems among Chinese migrant children [10]. On account of the massive number of migrant children and their poor psychological health status, exploring the underlying mechanisms in the association between acculturation stress and the mental health of Chinese migrant children is essential.

Acculturation stress can be defined as the stress that emerges from conflicts when individuals must adjust to a new culture of the host society [11,12]. According to the psychological acculturation theory [13], individuals’ psychological health changes to adapt to a new cultural situation. In dynamic migration, immigrants may confront tremendous challenges, such as poor language skills, limited educational opportunities [14], finite access to work [15], discrimination [16], and ethnic and cultural differences [17]. These migration-related stressors jeopardize the mental health of migrant populations. Based on the dual-factor model of mental health, negative indicators (e.g., anxiety, depression) and positive indicators (e.g., life satisfaction, subjective well-being) are related but different concepts, and both are worthy of examination [18,19].

Acculturation stress is widely acknowledged as a risk factor for individuals’ mental health [5,16,20,21]. In terms of its impact on negative mental health, Kartal and Kiropoulos (2016) supported that language barriers can be understood as a part of acculturation stress, which is associated with post-traumatic stress disorder (PTSD) and anxiety [22]. Another longitudinal research on multicultural youths demonstrated that acculturative stress can be regarded as a risk factor that might result in depression [23]. In the social context of China, Jiang and Dong (2020) document that discrimination from teachers is an explanatory variable for depression among migrant adolescents in China [5]. In addition, evidence shows that perceived discrimination from the host society, differences with natives, problems of obtaining legal status, and other kinds of acculturation stress lead to lower levels of psychological well-being [24], happiness [25], and life satisfaction [16]. However, discussions about the potential mechanisms between acculturation stress and mental health are rare, especially among Chinese migrant children.

According to the basic psychological needs theory [26], autonomy, relatedness, and competence are the three fundamental psychological needs [27]. Grounded in this theory, the need for autonomy refers to the experience of volition, initiative, and self-determination [28,29]. The need for relatedness involves the experience of closeness and feeling connected with others [28,30]. Competence concerns feelings of being effective and capable of achieving goals [28,31]. The recent theorizing of basic psychological needs theory illustrates that need frustration and a lack of need satisfaction is asymmetric [6,31,32] and that need frustration and need dissatisfaction are distinct concepts [33,34].

Satisfaction of these three basic psychological needs is beneficial for individuals’ psychological growth across various outcomes [26], including happiness [35], vitality [36], enthusiasm [37], self-esteem [38], and subjective well-being [39]. Yang et al. (2018) confirmed that high levels of basic psychological need satisfaction can promote life satisfaction based on a sample of 131 international students who studied abroad and adapted to the new environment [40]. By contrast, if these needs are frustrated, the risk of mental disorders and the possibilities of negative psychological consequences increase [6,30,31]. Recent empirical studies have indicated that need frustration is a significant predictor of depression [36], emotional exhaustion [37], and psychological ill-being [41].

Based on the basic psychological needs theory [26], social context might influence the extent to which individuals’ psychological needs can be satisfied or frustrated. A disempowering social context obstructs the individuals’ needs for autonomy, relatedness, and competence. In the current study, acculturation stress as a risk social contextual factor may hinder the satisfaction or enhance the frustration of basic psychological needs among migrant children. For example, those who are adjusting to a new culture can face acculturation stress such as a negative social context of school, which may undermine their connections with others and devaluate their motivation [42] and thereby frustrating psychological needs. Racial discrimination is also negatively associated with the satisfaction of psychological needs for autonomy in racial minority participants [43]. Additionally, Torres (2009) demonstrated that the satisfaction of the need for competence can mediate the relationship between perceived discrimination and depression among a sample of students from minority backgrounds [44]. Taken together, guided by the basic psychological needs theory, rational speculation is that acculturation stress may decrease the level of need satisfaction and increase the level of need frustration, which may further damage the mental health of migrant children.

In summary, this research field has certain gaps. First, the mediators between acculturation stress and mental health have not been investigated from a basic psychological needs theory perspective, and thus little is known regarding whether the satisfaction and frustration of basic psychological needs can mediate this association. Furthermore, most prior studies on acculturation focus on international migrants, while less attention is paid to children in the context of Chinese internal migration. Not only do transnational immigrants experience acculturation, internal migrants who relocate to distinct regions within a country also undergo social and cultural changes [45]. Similar to the adverse experiences faced by international migrants, Chinese migrant children face acculturation and deal with challenges such as prejudice and discrimination from local residents due to institutional and cultural barriers [46,47]. Nevertheless, China’s internal migrants vary from cross-border immigrants in several aspects. Compared with most western countries in which migration-related stress is based on the discrimination of ethnicity, the institutional constraints faced by China’s internal migrants are mainly generated by the rural–urban dual household registration system [48]. In general, the cultural differences between two regions within a country are smaller than those across countries. For instance, learning a new language is often regarded as a challenge for cross-national migrants. However, internal migrants in China hardly need to cope with language barriers because Mandarin is the official language and is widely used across the entire country. Thus, research on the population of internal migrant children in the specific social context of China is necessary.

On the basis of previous literature and its research gaps, the present study primarily focuses on two questions: (1) How does acculturation stress influence happiness and depression among Chinese migrant children? (2) How do need satisfaction and frustration mediate the effects of acculturation stress on migrant children’s mental health? As reviewed above, we propose the following research hypotheses.

**Hypothesis** **1** **(H1).** *Acculturation stress is positively associated with depression and negatively associated with happiness.*

**Hypothesis** **2** **(H2).** * Acculturation stress causes high levels of need frustration and low levels of need satisfaction, which in turn leads to high levels of depression and low levels of happiness.*

## 2. Methods

### 2.1. Participants

A sample of migrant children in Grades 4–9 in Kunming city from Southwest China was obtained using the multi-stage cluster random sampling method. In this study, migrant children referred to those under the age of 18 who were not born in Kunming (without Kunming household registration when they were born) and followed their legal guardians to move to the said city. Before the survey, a trained investigator described the purpose of the study and the ethical principles of voluntary participation, anonymity, and confidentiality. Participants acknowledged that withdrawal from the survey would not cause any adverse consequences. Signed informed consent forms were obtained from participants, their parents, and related school staff who agreed to participate in the study. The children filled out the questionnaires in classrooms, where research assistants stayed to answer any queries. Ethical approval is obtained from the research ethics committee of the affiliated university (No. SBRE-19-383). A total of 484 migrant children participated in the research. Participants’ ages ranged from 8–17 years (M = 11.65, SD = 1.61). The mean year of migration was 6.75 (SD = 3.11). Most of the participants were rural–urban migrants (71.1%), and a small percentage consists of urban–urban migrants (28.9%). Most of the participants (69%) migrated within the province, and the rest (31%) were originally from other provinces and moved to Kunming.

### 2.2. Measurements

#### 2.2.1. Acculturation Stress

The Acculturative Stress Inventory for Children [49] was adapted to assess the acculturation stress of migrant children. This scale contained two dimensions: perceived discrimination (e.g., Given my migration status, I feel others do not include me in a few of the things they do, games they play, etc.) and migration-related stress (e.g., I do not feel at home here in the host city). Responses ranged from 1 = strongly disagree to 5 = strongly agree. High scores indicated high levels of acculturation stress. The latent variable of acculturation stress was constructed using two indicators, namely, discrimination and migration-related stress. This scale has not been used in China and was translated into Chinese using a translation-back-translation procedure. Cronbach’s alpha was 0.86, indicating high reliability.

#### 2.2.2. Satisfaction and Frustration of Basic Psychological Needs

The Basic Psychological Need Satisfaction and Frustration Scale (BPNSFS) developed by Chen et al. (2015) was used to measure the mediators in this study [31]. BPNSFS consisted of 24 items and two subscales for need satisfaction and need frustration. Participants responded on a 5-point Likert scale that ranged from 1 = completely disagree to 5 = completely agree, with high scores indicating high need satisfaction or frustration. Need satisfaction was further divided into autonomy satisfaction, relatedness satisfaction, and competence satisfaction, while need frustration was further divided into autonomy frustration, relatedness frustration, and competence frustration. Cronbach’s alpha coefficients of the need satisfaction and need frustration subscales were 0.779 and 0.753, respectively.

#### 2.2.3. Happiness

The General Happiness Scale [50] was used to assess happiness. A sample item is “In general, I consider myself as”, with responses ranging from 1 = not a very happy person to 5 = a very happy person. The average score of the four items on this scale was calculated. High scores represented a high level of happiness. This instrument was translated into Chinese in this study and showed high validity and reliability. Cronbach’s alpha was 0.833, which demonstrated high internal consistency.

#### 2.2.4. Depression

Depression was assessed using the Chinese version of the Depression Subscale of the Brief Symptom Inventory (BSI) [51,52]. This scale consisted of six items to be answered on a 5-point scale from 1 = not at all to 5 = extremely. The average score of the six items was used to assess the depression level of children, with a high score indicating a high level of depression. Cronbach’s alpha was 0.899, which showed good reliability.

#### 2.2.5. Covariates

Sociodemographic variables in the survey were controlled as covariates in the present study, including gender (1 = male, 2 = female), age, migration time (years living in the inflow city), migration distance (1 = intra-provincial migration, 2 = inter-provincial migration), and migration pattern (1 = urban–urban migration, 2 = rural–urban migration).

### 2.3. Data Analysis

First, we carried out descriptive analyses on the means, standard deviations, and bivariate correlations between these variables. Then, structural equation modeling (SEM) was used following the two steps suggested by Anderson and Gerbing [53]. Confirmatory factor analysis was performed to test the measurement model, and then the hypothetical paths were validated in the structural model. To test the mediation effect, the bootstrap procedure was used with 5000 iterations. The indirect effect was identified as significant if zero was not included in the 95% bias-corrected confidence intervals (CI). Model fit was evaluated using the following indices: chi-square (χ^2^) statistics, comparative fit index (CFI), root mean square error of approximation (RMSEA), and standardized root mean square residual (SRMR). χ^2^ was sensitive to sample size and was not reliable as a basis for evaluating model fit. Therefore, χ^2^/df was used as a criterion. Overall, χ^2^/df < 3, CFI > 0.90, and RMSEA and SRMR < 0.08 indicated an acceptable model fit [54,55,56]. Descriptive analyses were performed in SPSS 25.0 (SPSS Inc., Chicago, IL, USA), and SEM was conducted using Mplus 8.0 (Muthén & Muthén, Los Angeles, CA, USA).

## 3. Results

### 3.1. Preliminary Analysis

Table 1 shows the descriptive statistics and correlation analysis for the study variables. The results showed that all of the correlation coefficients reached a significant level. Most of the coefficients ranged from 0.2 to 0.5, indicating small to moderate effects [57]. Acculturation stress was negatively correlated with need satisfaction (*r* = −0.219, *p* < 0.001) and happiness (*r* = −0.272, *p* < 0.001) and positively correlated with need frustration (*r* = 0.286, *p* < 0.001) and depression (*r* = 0.390, *p* < 0.001). Need satisfaction was positively related to happiness (*r* = 0.395, *p* < 0.001) and negatively related to depression (*r* = −0.310, *p* < 0.001). Need frustration was positively related to depression (*r* = −0.496, *p* < 0.001) and negatively related to happiness (*r* = −0.305, *p* < 0.001).

### 3.2. Measurement Model

The results showed that the measurement model fitted the data well: χ^2^ = 310.361, *df* = 113, χ^2^/df = 2.747, *p* < 0.001, CFI = 0.933, RMESA = 0.061, and SRMR = 0.044. The measurement model comprised three latent constructs (acculturation stress, basic psychological need satisfaction, and basic psychological need frustration). Specifically, the latent construct of acculturation stress included two first-order observed variables (discrimination with the loading of 0.986 and migration-related stress with the loading of 0.711), which were further constructed by the second-order items, with factor loadings ranging 0.513–0.822 and 0.570–0.688, respectively. The factor loadings for the latent constructs of basic psychological need satisfaction and frustration ranged 0.671–0.698, and 0.597–0.743, respectively. All of the standardized loadings of the observed variables reached a significant level on their corresponding latent variables (*p* < 0.001), indicating an effective representation.

### 3.3. Structural Model

**Figure 1:**
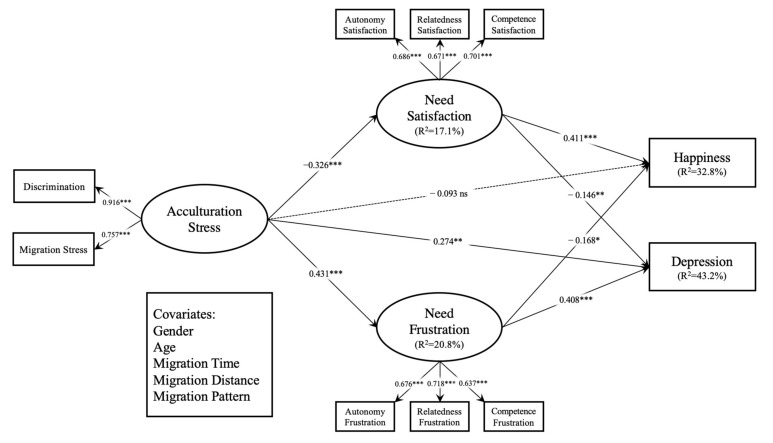
Structural equation model. Note. Standardized coefficients are shown. Non-significant paths are represented with dashed lines. *** *p* < 0.001, ** *p* < 0.01, * *p* < 0.05, ns = non-significant.

The structural model showed good model fit: χ^2^ = 527.987, *df* = 217, χ^2^/df = 2.433, *p* < 0.001, CFI = 0.911, RMESA = 0.055, and SRMR = 0.052. Table 2 and Figure 1 shows the results of the structural model. The coefficient of the direct path from acculturation stress to depression (*β* = 0.274, *p* < 0.01) was significant, but the direct path from acculturation stress to happiness was non-significant (*β* = −0.093, *p* > 0.05). Moreover, acculturation stress was negatively associated with need satisfaction (*β* = −0.326, *p* < 0.001) and positively associated with need frustration (*β* = 0.431, *p* < 0.001). Basic psychological need satisfaction was positively predictive of happiness (*β* = 0.411, *p* < 0.001) and negatively predictive of depression (*β* = −0.146, *p* < 0.01). The frustration of basic psychological needs was negatively related to happiness (*β* = −0.168, *p* < 0.05), and positively related to depression (*β* = 0.408, *p* < 0.001).

The significance of the mediating effects of need satisfaction and frustration was assessed using the bootstrap method. Table 3 shows the results of the bootstrap analysis, including standardized estimates, standard errors, *p*-values, and 95% confidence intervals (CIs). Acculturation stress had indirect effects on depression via the mediators of need satisfaction (*β* = 0.048, *p* < 0.05, 95% CI (0.014, 0.100)) and need frustration (*β* = 0.176, *p* < 0.001, 95% CI (0.114, 0.253)). The indirect effect of acculturation stress on happiness was also significant with need satisfaction (*β* = −0.134, *p* < 0.001, 95% CI (−0.205, −0.078)) and need frustration (*β* = −0.073, *p* < 0.05, 95% CI (−0.139, −0.016)) as the mediators. As zero was not included in the CIs, the mediating effects of need satisfaction and frustration were statistically significant. These results indicated that need satisfaction and frustration partially mediated the relationship between acculturation stress and depression and fully mediated the relationship between acculturation stress and happiness. The overall model accounted for 17.1%, 20.8%, 32.8%, and 43.2% of the variance in need satisfaction, need frustration, happiness, and depression, respectively.

## 4. Discussion

Based on a sample of migrant children in China, the present study aims to validate the mediating roles of need satisfaction and frustration in the associations among acculturation stress, happiness, and depression on the basis of basic psychological needs theory. Supporting the research hypotheses, the results indicate that a high level of acculturation stress results in a low level of need satisfaction and a high level of need frustration, which in turn lead to a high degree of depression and a low degree of happiness. The results are discussed as follows.

First, the results demonstrated that a high degree of acculturation stress is associated with a high level of depression. This result is consistent with the preceding literature that illustrates acculturation stress during migration is associated with children’s psychological disorders [22]. However, surprisingly, acculturation stress is not significantly associated with happiness. This finding differs from previous research that demonstrates acculturation stress has a negative influence on subjective well-being and happiness [24,25]. As a possible illustration, the cultural concept of turning suffering into blessing has long existed in China. Specifically, even though facing stress contributes to mental disorders, this cultural concept encourages migrant children to maintain positive attitudes and optimistic qualities, such as the pursuit of and the ability to experience happiness [58]. Therefore, acculturation stress is not a direct factor for migrant children’s happiness in the current study.

Moreover, need satisfaction and frustration are supported as mediators between acculturation stress and mental health from a dual asymmetric perspective. Acculturation stress affects happiness and depression because it impairs satisfaction or facilitates frustration of the basic psychological needs for autonomy, relatedness, and competence. This finding is consistent with previous studies demonstrating that need satisfaction is associated with well-being [39] and need frustration is linked with negative psychological consequences [41]. Chinese culture attaches importance to harmonious interpersonal relationships [59], and this cultural value prompts the Chinese to pay attention to how others evaluate them [60]. The distinct group membership caused by the household registration system leads to difficulties for migrants to receive positive feedback from local residents. The acculturation stress related to the migrant identity prevents migrant children from integrating into local society and leads to the frustration of their basic psychological needs, which further results in adverse psychological outcomes among this vulnerable group.

Furthermore, the present results suggest the necessity to measure need satisfaction and need frustration separately as distinct concepts that have different impacts on depression and happiness. The current study is in line with prior research that reports need satisfaction and frustration are not opposing sides of the same coin [32,33]. More crucial is that the relationship between need satisfaction and happiness is stronger than that between need satisfaction and depression, whereas the relationship between need frustration and happiness is weaker than that between need frustration and depression. This finding provides empirical support for the recent application of basic psychological needs theory in the Chinese context. Overall, the current study integrates the dual-factor model of mental health and basic psychological needs theory into a framework that fills the research gaps and extends current knowledge.

In spite of the above contributions, this study has several limitations that merit attention. First, this research is a cross-sectional study, which restricts the determination of causality among the variables. Therefore, the current findings need further validation through longitudinal research in the future. Second, the self-report measurement provides data that may not be adequately reliable because of existing measurement biases. Future studies can adopt multi-source measurements to overcome this limitation. Moreover, the present study does not examine the personality factors, which may have an impact on the association between acculturation stress and the mental health of migration children. Future research can further explore how culture-specific aspects of Chinese personality affect the psychological outcomes of migrant populations.

## 5. Conclusions

The current study validates the negative impact of acculturation stress on the mental health of Chinese migrant children and supports the mediating effects of basic psychological need satisfaction and frustration in such association. This study extends previous literature and contributes to current knowledge by validating the basic psychological needs theory in the context of Chinese internal migration. Based on the empirical findings, relevant practical implications are proposed for protecting the mental health of migrant children. First, social policies should be introduced to create an inclusive social atmosphere to reduce the institutional barriers of the migrant population. Certain measures can be adopted to reduce stereotypes, prejudices, and discrimination toward migrants, such as reducing school and residential segregation of migrant children and promoting the communication and mutual understanding between local residents and the migrant population. In addition, multi-element intervention programs can be implemented to promote children’s satisfaction and reduce their frustration with basic psychological needs for autonomy, relatedness, and competence. Child-focused interventions that directly aim to improve children’s self-awareness of need satisfaction can be designed and implemented [61]. In addition, cultivating need-supportive environments in children’s social systems is another vital strategy to promote the satisfaction of psychological needs. For example, in the family, migrant parents are encouraged to adopt autonomy-granting parenting to raise their children and allow them to self-determine their behaviors. In schools, teacher-facilitated need-supportive activities can be conducted to provide opportunities for children to satisfy their needs for autonomy, relatedness, and competence. Such activities include involving children in decision-making, promoting interactions among classmates, and building their self-confidence in challenging learning activities.

## Figures and Tables

**Table 1:** Descriptive statistics and bivariate correlations.

	M	SD	1	2	3	4	5
1. Acculturation stress	2.180	0.591	1				
2. Need satisfaction	3.642	0.708	−0.219 ***	1			
3. Need Frustration	2.475	0.687	0.286 ***	−0.257 ***	1		
4. Happiness	3.854	0.960	−0.272 ***	0.395 ***	−0.305 ***	1	
5. Depression	2.055	0.984	0.390 ***	−0.310 ***	0.496 ***	−0.545 ***	1

Note. *** *p* < 0.001

**Table 2:** Unstandardized and standardized path coefficients of the structural model.

	B	β	SE	*p*	R^2^
Need satisfaction					17.10%
Acculturation stress	−0.423	−0.326	0.120	<0.001	
Gender	−0.075	−0.251	0.031	0.018	
Age	0.004	0.011	0.020	0.857	
Migration time	0.006	0.058	0.006	0.327	
Migration distance	0.139	0.153	0.053	0.008	
Migration pattern	0.021	0.082	0.024	0.387	
Need frustration					20.80%
Acculturation stress	0.518	0.431	0.113	<0.001	
Gender	0.025	0.089	0.021	0.242	
Age	0.050	0.165	0.017	0.003	
Migration time	−0.003	−0.027	0.005	0.620	
Migration distance	−0.022	−0.026	0.048	0.647	
Migration pattern	−0.011	−0.044	0.019	0.572	
Happiness					32.80%
Acculturation stress	−0.196	−0.093	0.165	0.237	
Need satisfaction	0.670	0.411	0.117	<0.001	
Need frustration	−0.296	−0.168	0.117	0.012	
Gender	−0.115	−0.238	0.036	0.001	
Age	−0.014	−0.026	0.026	0.595	
Migration time	0.015	0.093	0.007	0.038	
Migration distance	−0.070	−0.047	0.067	0.293	
Migration pattern	0.048	0.116	0.030	0.105	
Depression					43.20%
Acculturation stress	0.572	0.274	0.194	0.003	
Need satisfaction	−0.235	−0.146	0.090	0.009	
Need frustration	0.710	0.408	0.126	<0.001	
Gender	−0.044	−0.092	0.032	0.171	
Age	0.034	0.064	0.023	0.144	
Migration time	0.002	0.015	0.007	0.751	
Migration distance	−0.018	−0.012	0.062	0.772	
Migration pattern	−0.015	−0.036	0.027	0.580	

Note. B: unstandardized path coefficient; β: standardized path coefficient; SE: standard error; *p*: significance level.

**Table 3:** Direct, indirect, and total effects.

	β	SE	*p*	95% CI	
				Lower	Upper
Effects from acculturation stress to depression					
Direct effect: Acculturation stress→Depression	0.274	0.074	<0.001	0.132	0.427
Indirect effect: Acculturation stress→Need satisfaction→Depression	0.048	0.020	0.019	0.014	0.100
Indirect effect: Acculturation stress→Need frustration→Depression	0.176	0.035	<0.001	0.114	0.253
Total effect	0.497	0.058	<0.001	0.384	0.612
Effects from acculturation stress to happiness					
Direct effect: Acculturation stress→Happiness	−0.093	0.072	0.196	−0.240	0.049
Indirect effect: Acculturation stress→Need satisfaction→Happiness	−0.134	0.033	<0.001	−0.205	−0.078
Indirect effect: Acculturation stress→Need frustration→Happiness	−0.073	0.031	0.020	−0.139	−0.016
Total effect	−0.299	0.062	<0.001	−0.422	−0.174

Note. β: standardized coefficient; SE: standard error; *p*: significance level; CI: confidence interval.

## Data Availability

The data presented in this study are available on request from the corresponding author.
